# Effects of Emergence Time and Early Social Rearing Environment on Behaviour of Atlantic Salmon: Consequences for Juvenile Fitness and Smolt Migration

**DOI:** 10.1371/journal.pone.0119127

**Published:** 2015-03-06

**Authors:** Martin H. Larsen, Jörgen I. Johnsson, Svante Winberg, Alexander D. M. Wilson, David Hammenstig, Per-Ove Thörnqvist, Jonathan D. Midwood, Kim Aarestrup, Erik Höglund

**Affiliations:** 1 National Institute of Aquatic Resources, Section for Freshwater Fisheries and Ecology, Technical University of Denmark, Silkeborg, Denmark; 2 Department of Biological and Environmental Sciences, University of Gothenburg, Gothenburg, Sweden; 3 Department of Neuroscience, Uppsala University, Uppsala, Sweden; 4 Fish Ecology and Conservation Physiology Laboratory, Department of Biology and Institute of Environmental Science, Carleton University, Ottawa, Ontario, Canada; 5 National Institute of Aquatic Resources, Section for Aquaculture, Technical University of Denmark, Hirtshals, Denmark; 6 Research Secretariat, University of Agder, Kristiansand, Norway; The Ohio State University, UNITED STATES

## Abstract

Consistent individual differences in behaviour have been well documented in a variety of animal taxa, but surprisingly little is known about the fitness and life-history consequences of such individual variation. In wild salmonids, the timing of fry emergence from gravel spawning nests has been suggested to be coupled with individual behavioural traits. Here, we further investigate the link between timing of spawning nest emergence and behaviour of Atlantic salmon (*Salmo salar*), test effects of social rearing environment on behavioural traits in fish with different emergence times, and assess whether behavioural traits measured in the laboratory predict growth, survival, and migration status in the wild. Atlantic salmon fry were sorted with respect to emergence time from artificial spawning nest into three groups: early, intermediate, and late. These emergence groups were hatchery-reared separately or in co-culture for four months to test effects of social rearing environment on behavioural traits. Twenty fish from each of the six treatment groups were then subjected to three individual-based behavioural tests: basal locomotor activity, boldness, and escape response. Following behavioural characterization, the fish were released into a near-natural experimental stream. Results showed differences in escape behaviour between emergence groups in a net restraining test, but the social rearing environment did not affect individual behavioural expression. Emergence time and social environment had no significant effects on survival, growth, and migration status in the stream, although migration propensity was 1.4 to 1.9 times higher for early emerging individuals that were reared separately. In addition, despite individuals showing considerable variation in behaviour across treatment groups, this was not translated into differences in growth, survival, and migration status. Hence, our study adds to the view that fitness (i.e., growth and survival) and life-history predictions from laboratory measures of behaviour should be made with caution and ideally tested in nature.

## Introduction

In recent years, it has become increasingly recognized that individuals within a population can differ consistently in suites of correlated behavioural traits across time and context. Such individual variation has been reported in a wide variety of animal taxa including fishes [1,2], birds [3,4], mammals [5,6], reptiles [7], amphibians [8] as well as a range of invertebrates [9–11], with the terms temperament, personality, and behavioural syndrome being used to describe this phenomenon [12–15]. In addition, the term stress coping style, characterized along a proactive–reactive continuum, is used for describing individual variation in both behavioural and physiological responses to stress [16]. To date, the majority of studies regarding such individual variation have focused on adult animals, but recently questions have arisen about its existence in early ontogeny and its relation to life-history traits [8,17].

In salmonids, eggs are deposited by the female in gravel nests on the stream bottom. After hatching, the larvae remain within the gravel and feed endogenously on their yolk sac, whereupon they emerge from the spawning nest and start defending feeding territories. The time to emerge from the gravel may vary by several weeks among individuals in the same spawning nest [18,19]. Those that emerge first from the nest are generally considered to have a competitive advantage over later conspecifics because of early access to profitable feeding territories [20–23]. However, this benefit may be offset by costs such as increased predator exposure [24]. These conflicting selective pressures are believed to play an important role in maintaining variability in larval developmental rate in the wild [22].

Several studies have shown that the variation in the timing of spawning nest emergence is related to behavioural traits expressed by the individual [25–28]. For example, Metcalfe and Thorpe [25] demonstrated that early emerging Atlantic salmon (*Salmo salar*) fry were more aggressive and had a higher probability of becoming socially dominant compared to those emerging late. Inherited components underlying this relationship have been demonstrated in two strains of rainbow trout (*Oncorhynchus mykiss*) selected for stress responsiveness (i.e., post stress plasma cortisol; see [29]). The strain exhibiting low stress responsiveness (LR), characterized by having an increased propensity for boldness and social dominance, also showed an earlier emergence time compared to the shy and less socially competitive high stress responsive (HR) strain [28]. These findings indicate a relationship between stress coping styles and timing of spawning nest emergence in salmonid fishes [25,28].

Although consistent individual variation in behaviour is now well documented in several fish species (reviewed in Conrad et al. [1]), remarkably little is known about whether and to what extent behavioural traits expressed early in ontogeny influence fitness (e.g., growth and survival) and life-history trajectories. In addition, studies of behavioural trait variation have mainly been conducted under laboratory conditions and the ecological consequences of consistent individual differences in behaviour for fish in the wild remains unclear [30,31]. Salmonids represent excellent candidates for addressing such questions as they usually exhibit extensive variation in behavioural and life-history patterns such as age at sexual maturity and sea migration [32]. While some studies of juvenile salmonids have demonstrated that fitness and life-history strategies may be linked to individual differences in behavioural traits [25,33–35], others have been unable to document such relationships [36,37]. Moreover, it has been suggested that behavioural characteristics associated with spawning nest emergence can affect growth and timing of smoltification (seawater adaptation) in salmonids reared under hatchery conditions [25,38]. However, it remains unknown if such relationships persist in natural habitats, where environmental complexity is higher and there are more dynamic ecological factors influencing selection such as competition, predation, and resource availability.

A study on the HR and LR rainbow trout strains also suggest effects of the social environment on fitness-related traits [39]. When co-reared, the more bold and aggressive LR strain outgrew the HR strain, an effect not seen when these two strains were reared separately. Additionally, several studies suggest that behavioural traits may be plastic and sensitive to social conditions [40,41]. Studies on rainbow trout, for example, show that previous conspecific experience may alter the degree of boldness and aggression of individuals [41,42]. Magnhagen and Staffan [40] demonstrated that individual young-of-the-year perch (*Perca fluviatilis*) can adjust boldness and exploration tendency relative to their social group. However, there is a shortage of studies that elucidate the effects of social environment early in ontogeny in shaping an individual’s behavioural trait composition and its potential for affecting fitness and life-history trajectories.

Knowledge of how individuals with different behavioural traits perform in nature is crucial for incorporating the framework of consistent behavioural variation into management practices [1,30]. This requires that high throughput methods for characterizing behavioural trait composition must be developed. As mentioned previously, there seems to be a relationship between emergence time from spawning nests and the behavioural profile of an individual. Therefore, sorting salmonid fishes according to larval developmental rate in the hatchery may offer such a method for behavioural characterization. Potentially, this will generate knowledge about the linkage between larval developmental rate and behavioural trait compositions, which may be applied in restocking strategies. In this respect, it is equally important to understand the potential impacts of social environments on behavioural expression during the early rearing phase before releasing the hatchery fish into the wild.

In the present study, our primary objectives were to investigate if the relationship between behavioural traits and the time of emergence is affected by the social rearing environment, and to investigate whether trait compositions expressed in the hatchery are related to growth, survival, and migration status in natural environments. To assess this, Atlantic salmon fry were sorted with respect to time of emergence into three groups: early (first one-third), intermediate (middle one-third), and late (last one-third). To investigate social effects on behavioural traits, these groups were reared separately or in co-culture for four months. After this, the behaviour profile of individuals with different emergence time and rearing environment where characterized by a basal locomotor activity test [34], a novel object test [43], and a net restraining test [44]. Following behavioural characterization, fish were released into a closed near-natural experimental stream to examine the potential effects of differences in emergence time and previous rearing environment on fitness (i.e., growth and survival) and smolt migration.

## Materials and Methods

### Ethics statement

This study was carried out in strict accordance with the recommendations of the Swedish Board of Agriculture and conformed to the national guidelines of Sweden. All animal procedures were approved by the Ethical Committee for Animal Research in Gothenburg (Dnr 8-211). All efforts were made to minimize animal handling and stress.

### Experimental fish and housing conditions

The fish used in the present study were first-generation offspring (F1) of Atlantic salmon originating from the River Dalälven in central Sweden. Since the construction of a power plant in 1915 in Älvkarleby, located about 10 km from the river mouth to the Gulf of Bothnia, anadromous fish have been prevented from completing their upstream migration to spawning grounds beyond the dam. To compensate for the loss of natural production of Atlantic salmon, mature adults are caught annually in the river and artificially crossed in order to create supportive stocks. The offspring are hatchery-reared and released as 1- and 2-year-old smolts in the river during spring. Although Atlantic salmon spawn naturally in the river below the dam, wild smolt production in this system is minimal, representing less than 1% of total hatchery supplementation [45]. In this study, the parental fish were caught in a permanent fish-trap situated at the upper end of Kungsådran (a 1.6 km long branch of River Dalälven) during the autumn of 2011. The fish were transported to the Fishery Research Station in Älvkarleby and kept in indoor concrete ponds. On the 7^th^ of November 2011, eggs from 24 females (73–104 cm in total length) were stripped and fertilized with the milt from 24 males (71–107 cm in total length). Eggs from each female were fertilized by a haphazardly chosen male and incubated separately in hatching trays. At 141 day degrees post fertilization (DDF), eyed eggs were placed into eight artificial spawning nests, which allowed fry to be sorted with respect to emergence time. Each spawning nest contained 600 eggs originating from three different females (i.e., 200 eggs from each female). The spawning nests consisted of three parts: a flat holding tray with golf balls as bottom substrate to mimic natural gravel, an emergence route, and a collection tank (see [27]). The nests were continuously provided with water from River Dalälven at ambient temperature (mean: 7.1°C; range: 2.4–15.7°C) and the photoperiod followed natural day-light cycles. After hatching, the larvae laid dormant and undisturbed until they reached an appropriate developmental stage and started to emerge from the artificial gravel in search of food. During the period of emergence, the fry were transported downstream by a water current to the emergence route which ended up in the collection tank. Fry were removed daily from the collection tanks and divided into three different emergence groups: early (first 200 fish), intermediate (middle 200 fish), and late (last 200 fish). The emergence period was initiated on the 4^th^ of May 2012 (at 338 DDF) and after 24 days all individuals had left the spawning nests (at 611 DDF). The overall mortality rate was low (4%) during this period. Fry from the eight spawning nests were held in one of three tanks based on their emergence time (i.e., early, intermediate, and late). After all fry had emerged, individuals from each emergence group were collected and haphazardly divided among four replicate tanks (300 L, 110 × 110 × 25 cm) such that there were 370 fish in each tank. The fry were fed commercial trout pellets (Aller Performa, Aller Aqua, Denmark) with an automatic feeder.

Between the 12^th^ and14^th^ of June 2012, the fish were marked with different colored visible implant elastomers (VIE) according to emergence fraction (fluorescent red, green, and blue; Northwest Marine Technology, Washington, USA). Fish were anaesthetized (benzocaine 20 mg L^−1^) and placed on a wet tissue under a stereomicroscope. The marker was injected in the adipose eyelid tissue behind the eye at a shallow angle using a 29 gauge needle. When applied properly, VIE-tagging has no measurable effects on growth and survival of small salmonids [46]. A haphazardly collected subsample of fish from each of the three emergence groups was preserved in ethanol (80%) to determine length and body mass at time of VIE-tagging (n = 30 for each emergence group). Overall, the average fork length (FL) and mass of these preserved individuals was 30.6 ± 0.171 (SE) mm and 0.19 ± 0.004 (SE) g, respectively, and no differences were found among the emergence groups (generalized linear model; length: F = 0.926, df = 2, p = 0.400; mass: F = 1.077, df = 2, p = 0.345).

To investigate social rearing effects of differences in emergence time, fish with different emergence times were reared either separately (mono-culture) or mixed (co-culture) for approximately four months post-emergence. This resulted in four different rearing groups (early, intermediate, late, or mixed emergence time) that were each replicated three times. The mixed group replicates each consisted of 300 individuals (100 individuals from each of the three emergence groups) and the replicates for the separately reared groups each consisted of 300 individuals per emergence group. Fish from each emergence group were randomly selected from the four replicate tanks in equal numbers. Each rearing group was reared in three replicate tanks (300 L, 110 × 110 × 25 cm; 12 tanks in total) supplied with river water (Dalälven) and kept under natural photoperiod and temperature (mean: 15.1°C; range: 5.9–20.0°C) conditions. Fish were fed daily during daylight hours with commercial trout pellets at a ratio equivalent to 2% of body mass using automatic feeders. The pellet size was changed in accordance to the manufacturer’s recommendations as the fish increased in size.

### Behavioural tests

**Fig 1:**
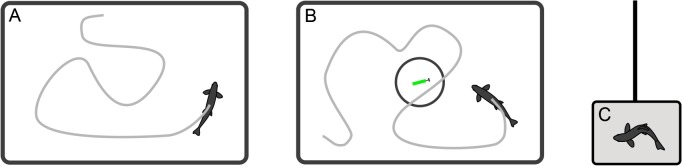
Schematic representation of the experimental set-up for the measurements of individual behavioural traits. (A) basal locomotor activity test, (B) novel object test, and (C) net restraining test. The novel object zone is indicated by the circle. The different elements are not to scale.

Between the 11^th^ and 26^th^ of October 2012, the individual behaviour of 20 fish from each of the six treatment groups consisting of the three emergence groups (early, intermediate, and late) reared under two conditions (mono-culture and co-culture) was scored (i.e., 120 fish in total). The 20 individuals in each treatment group were haphazardly collected from each of the three replicate tanks in roughly equal numbers (i.e., between 6–7 fish from each tank). However, to avoid potential adverse effects of PIT-tagging (see below) on growth and survival only fish above 85 mm FL were included in the experiment [47]. Behavioural trials (n = 15) occurred in batches of eight fish. Each fish was transferred to one of eight experimental tanks (opaque white plastic tanks, 55 × 36 cm, water level 6.5 cm) in the afternoon (at 17:00) prior to the day of behavioural observations. Hence, fish could not see one another during the behavioural tests. The tanks were arranged in two rows of four units which were positioned underneath two video cameras to allow simultaneous recording. To avoid potential tank effects, we randomized allocation of the fish from the different treatment groups between the experimental tanks. In addition, at least one fish from each treatment group was represented during each trial to minimize time effects. The next day, the behaviour of each fish was scored in each of the following contexts: (1) a basal locomotor activity test (adapted from [34,48]), (2) a novel object test (adapted from [34,41,43]), and (3) a net restraining test (adapted from [44,49,50]; Fig. 1). These assays were chosen because they characterize important aspects of the behaviour of juvenile Atlantic salmon, such as basal activity pattern, boldness, and their escape response. All behavioural tests were carried out between 09:00 am and 12:15 pm on the day following transfer to the experimental set-up and the timing of all observations was similar among trials. The entire experimental arena was surrounded by an observation blind constructed from green tarpaulin, ensuring that fish were not disturbed during behavioural observations. The experimental tanks were supplied with flow-through river water at a flow rate of 2.6 L min^−1^, corresponding to approximately 12 cycles per hour. The water temperature ranged between 5–8°C and the tanks were exposed to a 9 h light:15 h dark cycle with day break set at 07:00 am. Illumination was provided by two lamps (Walimex Daylight 1000, Burgheim, Germany) and the light intensity was 160 lux. Experimental tanks were cleaned with ethanol (95%) and subsequently flushed with river water between each trial. Fish were not fed after being moved to the behavioural observation tanks.

#### Basal locomotor activity test

In the first test, the undisturbed swimming pattern (locomotor activity) of the juvenile Atlantic salmon was video recorded for 30 min in the experimental tanks (Fig. 1). Based on interpretation of the videos, the following information for each individual was recorded: average swimming velocity (cm/s), total time (s) with velocity greater than 1 cm/s (i.e., total time active), and maximum recorded velocity (cm/s).

#### Novel object test

Following the basal locomotor activity test, the tendency of the fish to explore a novel object was measured (Fig. 1). The novel object test has been widely used in behavioural research to assess boldness in fish (e.g., [41,43]). The object consisted of a bright-green rawlplug (3.5 cm long and 0.8 cm in diameter) with a stainless screw (served as ballast). The object were attached to a transparent fishing line and gently lowered down in the center of the experimental tank. The 30 min observation period started after the novel object was lowered to the bottom of the tank. A radius of 5 cm around the novel object was defined as the novel object zone. The following behaviours were recorded for subsequent analyses: latency to enter the zone (s), total number of entries into the zone, total time spent within the zone (s), and average distance to the zone (cm). Entrance into the novel object zone was defined when the center-point of the fish was inside the area. The latency was set to 1800 s if the fish did not enter the zone. Any fish that was positioned in the novel object zone prior to the object being lowered was excluded from further analysis of boldness (n = 1).

#### Net restraining test

The net restraining test has been shown to correlate with physiological and behavioural traits in different fish species, and it has been previously used to distinguish individuals with contrasting stress coping styles; the proactive and reactive stress coping styles [44,49,50]. Each fish was transferred from the experimental tanks following the novel object test to an aerated bucket (15 L) covered with a lid. After a 30 min resting period, the fish was gently poured from the bucket into a net, mounted on a retort stand at an angle of 45°. While in the net, the total time spent on escape attempts (s) was recorded for 60 s. An escape attempt was defined as a tail beat. If for some reason a test fish could not be transferred within a standardized 10 s protocol for the restraining test, it was excluded from further analyses of escape behaviour (n = 3).

After completion of the net restraining test, fish were anaesthetized and surgically implanted with 12.5 mm PIT tags (ISO FDX-B, 134.2 kHz, diameter 2.1 mm; Biomark, Boise, Idaho, USA) to allow for individual recognition. The PIT tag was inserted into the peritoneal cavity through an approximately 2 mm long ventrolateral incision made slightly anterior to the muscle bed of the left pelvic fin. The tagging incision was left to heal without closure [51]. Fork length and mass of the fish were measured to the nearest 1 mm and 0.1 g, respectively. There were no significant differences in length and mass among fish scored for behaviour in the six treatment groups (generalized linear model; length: F = 0.178, df = 5, p = 0.970; mass: F = 0.378, df = 5, p = 0.863; Table 1).

**Table 1:** Fork length and mass of the Atlantic salmon (*Salmo salar*) among experimental treatments (n = 20 for each treatment) measured directly after the behavioural assays.

Treatment	Length (mm)		Mass (g)	
	Mean ± SE	Range	Mean ± SE	Range
**Mono-culture**				
Early	99.5 ± 1.06	91–108	10.5 ± 0.31	7.9–13.6
Intermediate	99.8 ± 1.18	91–110	10.7 ± 0.33	7.8–14.0
Late	98.3 ± 0.97	89–105	10.1 ± 0.27	7.5–11.7
**Co-culture**				
Early	99.3 ± 1.15	88–110	10.2 ± 0.42	6.9–15.0
Intermediate	99.3 ± 1.29	90–108	10.4 ± 0.40	7.4–13.5
Late	99.2 ± 1.19	89–112	10.3 ± 0.38	7.6–14.8

Following PIT-tagging, the fish scored in the behavioural trials were returned to new holding tanks until they were released in an experimental stream (see below). Individuals with early, intermediate, and late emergence time reared in mono-culture were kept in three separate tanks (200 L, 100 × 100 × 20 cm) and the mixed emergence time group was reared in a larger tank (600 L, 100 × 200 × 30 cm). Hence, the density of fish from the different treatments groups was similar in terms of number fish per cubic meter. All fish were in good condition after tagging and no tags were rejected in the tanks.

### Performance in the stream

On the 7^th^ of November 2012, the 120 fish scored in the behavioural trials were introduced into an experimental stream in Älvkarleby to compare growth, survival, and migration status (resident or migrant) of the different treatment groups under near-natural conditions (see [52] for a schematic view of the experimental stream). A tube supplies the stream with river water from the nearby hydro-electric dam in River Dalälven. The experimental stream has a length of 110 m and a total area of 345 m^2^. Wolf traps are situated at the upper and lower end of the stream and all water is running through the traps. A system of stainless gratings leads the fish to a collection tank (mesh size: 1 × 1 mm). Hence, no Atlantic salmon can escape from the stream, nor can any fish invade the system. The experimental stream consists of four pools with three riffles in between. The maximum water depth of the pools ranges from 0.8 to 1.2 m. The bottom substratum varies from mud, through coarse sand, to gravel, and larger stones. During the experiment the adjustable water flow was set to a maximum of 150 L s^−1^ and the average water temperature in the stream was 2.7°C (range: 0.1–18.0°C).

Fish were haphazardly released in equal numbers into the two middle pools of the stream. The density of Atlantic salmon in the stream corresponded to 35 individuals per 100 m^2^. This density is within the range of natural population densities of juvenile Atlantic salmon in the region [53]. Throughout the entire experiment, the downstream and upstream trap was inspected daily for the presence of Atlantic salmon. No fish were caught in the upstream trap. Length and mass of descending fish were measured and their PIT tag number was recorded using a handheld reader (Biomark 601, Boise, Idaho, USA). On the 4^th^ of June 2013, approximately seven months after fish were released, the experimental stream was drained slowly to recover surviving fish that remained in the system. All fish were measured, weighed, and identified. Fish only had access to naturally occurring food items, and were exposed to natural predation from American mink (*Mustela vison*) and gray heron (*Ardea cinerea*). For information on important prey taxa in the stream, see Johnsson et al. [52].

### Data handling and statistics

#### Video analyses

Recorded videos of basal locomotor activity and novel object test were analyzed with the video-tracking software EthoVision XT 10 (Noldus Information Technology, Wageningen, Netherlands) at a recording rate of 5 frames per second. This software distinguishes fish from their ambient background on the basis of their greyscale/brightness values. Center-point detection of the fish was chosen as the default setting in all tests and tracks were smoothed using the LOWESS algorithm. After the analysis was complete, the tracks were visually inspected for artifacts. Subject loss due to misdetections by video-tracking software was <0.7%. The recorded escape response behaviour of the Atlantic salmon in the net restraining test was analyzed manually using a media player (SMPlayer for windows version 0.8.6). The video recordings were played at 0.5 × normal speed during analyses.

#### Behaviour

Separate principal component analyses (PCAs) were used to summarize the behavioural measures for the basal locomotor activity test (i.e., average swimming velocity, total time active, and maximum recorded velocity) and the novel object test (i.e., latency to enter the zone, total number of entries into the zone, total time spent within the zone, and average distance to the zone). Each PCA resulted in one main principal component (PC) with eigenvalue greater than one (Kaiser-Guttman criterion), further referred to as PC activity and PC boldness respectively. The correlation matrix was used to check multicollinearity of variables. Kaiser-Meyer-Olkin (KMO) test for sample adequacy was greater than 0.6 and the Bartlett’s test of sphericity was significant for all tests (p < 0.0001) [54]. Only variables with loadings >|0.4| were considered to be important in constructing a principal component (sensu [54]). The extracted component scores (i.e., PC activity and PC boldness) were used as representative behavioural scores in subsequent analyses. To analyze effects of emergence time, rearing environment, and fish length on PC activity and PC boldness, two separate GLMs (generalized linear model) with Gaussian distribution and identity link function were used according to the following model (M1):

Responsevariable=intercept+emergencetime+rearingenvironment+length+(emergencetime×rearingenvironment)

with emergence time (early, intermediate, and late) and rearing environment (mono-culture and co-culture) as fixed factors. Fork length measured directly after the behavioural tests was included as covariate to correct for potential effects of body size. M1 was also used to analyze the duration of escape attempts in the net restraining test. Across experimental treatment groups, correlations between PC activity, PC boldness, and duration of escape attempts during net restraining were assessed using non-parametric Spearman rank correlations because these variables were not all normally distributed.

#### Performance in the stream

Survival rate in the experimental stream was calculated as the number of fish recovered in the stream at the end of the experiment plus the fish caught in the downstream trap divided by the total number of fish released in the stream multiplied by 100. The percentage of fish that migrated was calculated as number of fish caught in the downstream trap divided by the total number of fish released in the stream multiplied by 100. GLMs with binomial distribution and logit link function, also known as logistic regression, were used to model survival and downstream migration probabilities in the experimental stream, including the same variables as in M1. Likelihood ratio tests (LRT) were used to evaluate overall model significance. Specific growth rate (SGR; g day^−1^) for mass was calculated according to the following formula:

$$\text{SGR}=({\text{log}}_{\text{e}}{\text{M}}_{\text{f}}-{\text{log}}_{\text{e}}{\text{M}}_{\text{i}})\;\times \;{\text{t}}^{-1}$$

where M_i_ and M_f_ are the initial and final mass (g), respectively, and t is the time in days. A GLM with Gaussian distribution and identity link function was used to model the average SGR for Atlantic salmon among the treatment groups according to M1. Effects of PC activity, PC boldness, and duration of escape attempts during net restraining on the probability of survival and migration in the stream were investigated using separate GLMs with binomial distribution and logit link function. Finally, the correlation of SGR with PC activity, PC boldness, and duration of escape attempts was assessed using Pearson’s correlation.

#### Statistical software, data exploration, and model assumptions

Initial data exploration was based on the protocol in Zuur et al. [55]. Cleveland dotplots, boxplots, and multipanel scatter plots were used to identify outliers and to examine relationship between variables. Assumption of homogeneity of variance and normal distribution for the GLM models was ensured by visual inspection of residual plots, and Cook’s distance was calculated to detect any values exerting extreme influence on the model fit. PC activity and PC boldness required a rank transformation to satisfy the assumption of normality. The interaction between emergence time and rearing environment was excluded from the final model if p > 0.05. In case of a significant main term, Tukey honestly significant difference (HSD) test was employed to investigate differences among treatment groups. All statistical analyses were performed in R 3.0.1 (R Development Core Team 2013) and SPSS 20.0 (IBM Corporation, Armonk, NY, USA). Variation in association with recorded mean values is given as standard error throughout. Statistical significance for all analyses was assessed at an alpha level of 0.05.

## Results

### Behaviour

Separate PCAs on the behavioural measures for the basal locomotor activity and novel object test each extracted one main principal component, further referred to as PC activity and PC boldness respectively (Table 2 and 3). Average swimming velocity, total time active, and maximum velocity contributed to PC activity, with high positive loadings for all variables (Table 3); individuals that scored highly on this component had high levels of activity. PC boldness reflects the degree of boldness displayed by an individual and it was dominated by three variables; number of entries and duration of visits to the novel object zone were positively associated with one another, but negatively associated with the latency to enter the zone (Table 3). Therefore, high values on this component indicate bolder individuals. Average scores for PC activity and PC boldness were not affected by emergence time, rearing environment, or their interaction (GLM; F ≤ 2.110, df = 1–2, p ≥ 0.126). However, smaller fish generally had higher scores for PC boldness than larger conspecifics (GLM; F = 5.164, df = 1, p = 0.025).

**Table 2:** Descriptive values (mean ± SE) for scores of behavioural traits in each of three behavioural contexts: (1) basal locomotor activity test, (2) novel object test, and (3) net restraining test.

	Mono-culture			Co-culture		
Context/score	Early	Intermediate	Late	Early	Intermediate	Late
**Basal locomotor activity test**						
Average velocity (cm/s)	2.4 ± 0.21 (20)	1.5 ± 0.29 (20)	1.9 ± 0.26 (20)	2.0 ± 0.43 (20)	2.0 ± 0.32 (20)	2.1 ± 0.24 (20)
Total time active (s)	651.9 ± 52.77 (20)	404.0 ± 73.86 (20)	514.8 ± 66.76 (20)	488.9 ± 87.15 (20)	537.4 ± 80.13 (20)	563.9 ± 62.82 (20)
Maximum velocity (cm/s)	12.8 ± 0.61 (20)	12.7 ± 1.36 (20)	12.1 ± 1.24 (20)	12.2 ± 0.92 (20)	12.2 ± 0.82 (20)	12.5 ± 0.74 (20)
**Novel object test**						
Total time in the zone (s)	3.8 ± 1.39 (20)	5.0 ± 2.30 (20)	4.5 ± 1.73 (20)	10.6 ± 6.52 (19)	3.4 ± 1.46 (20)	4.6 ± 1.46 (20)
Number of zone entries	1.8 ± 0.52 (20)	1.6 ± 0.51 (20)	1.7 ± 0.60 (20)	2.1 ± 0.73 (19)	1.8 ± 0.88 (20)	1.8 ± 0.54 (20)
Latency to enter the zone (s)	1209.0 ± 154.71 (20)	1385.1 ± 119.54 (20)	1400.6 ± 136.38 (20)	1198.1 ± 140.80 (19)	1379.1 ± 141.38 (20)	1185.6 ± 157.09 (20)
Average distance to the zone (cm)	15.3 ± 1.03 (20)	17.0 ± 0.84 (20)	16.0 ± 1.00 (20)	14.2 ± 1.14 (19)	17.1 ± 0.69 (20)	14.7 ± 0.99 (20)
**Net restraining test**						
Total escape time (s)	17.5 ± 1.99 (20)	22.7 ± 2.21 (18)	21.9 ± 2.30 (19)	18.8 ± 2.21 (20)	23.7 ± 2.32 (20)	21.8 ± 1.79 (20)

Sample sizes are given in parentheses.

**Table 3:** Results of principal component analyses (PCAs) on behavioural traits in each of two behavioural contexts: (1) basal locomotor activity test, and (2) novel object test.

Principal components	PC
**PC activity**	
Behaviour	
Average velocity (cm/s)	**0.96**
Total time active (s)	**0.97**
Maximum velocity (cm/s)	**0.81**
Eigenvalue	2.51
% variance explained	83.58
**PC boldness**	
Behaviour	
Total time in the zone (s)	**0.80**
Number of zone entries	**0.87**
Latency to enter the zone (s)	**−0.89**
Average distance to the zone (cm)	−0.23
Eigenvalue	2.22
% variance explained	55.58

Loadings, eigenvalues, and explained variance (%) were calculated separately for behaviours describing basal activity (PC activity, n = 120) and boldness (PC boldness, n = 119). Loadings with absolute value above 0.4 are shown in bold.

**Fig 2:**
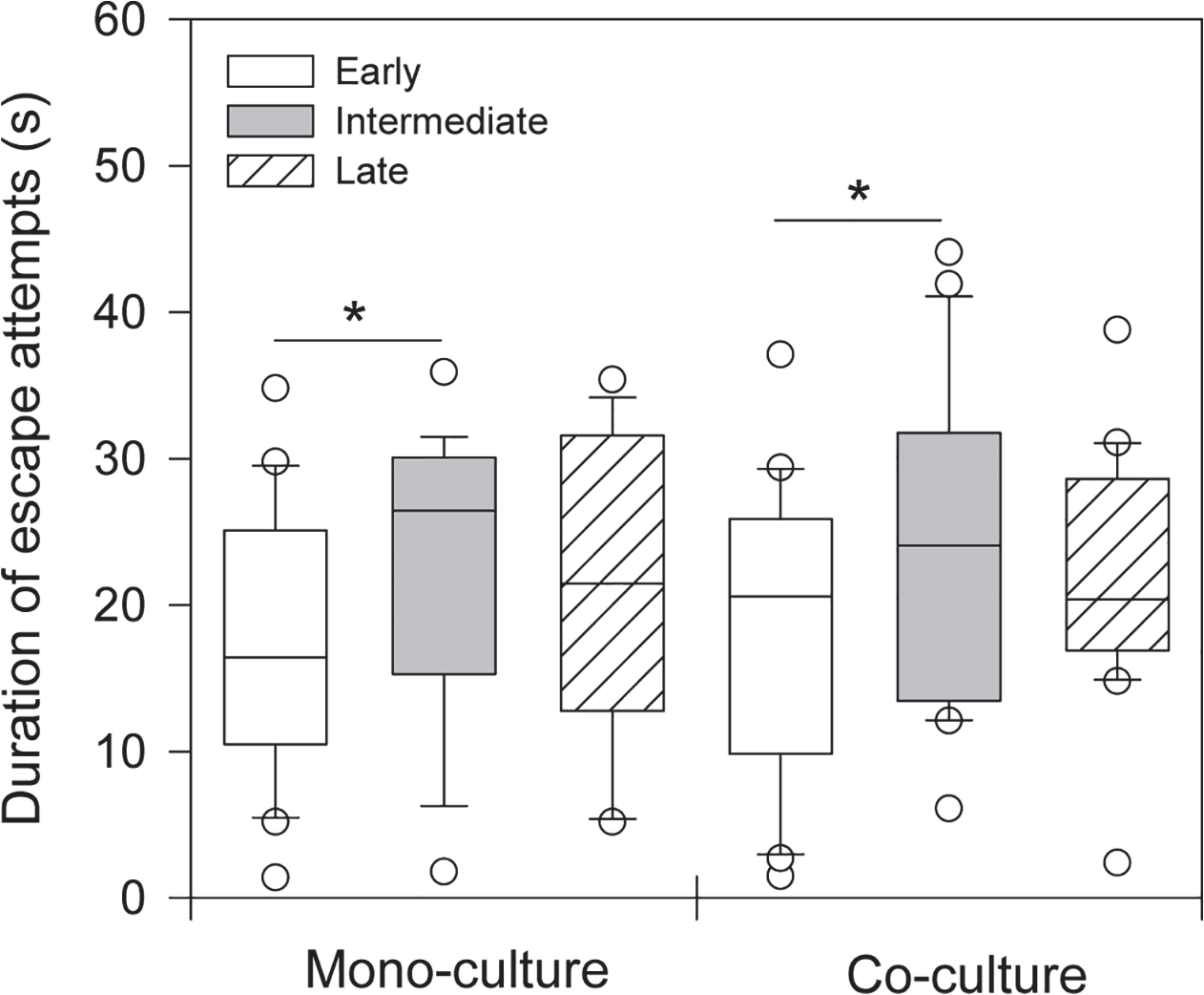
Duration of escape attempts (s) of Atlantic salmon (*Salmo salar*) during a net restraining test. Fish were sorted according to emergence time from artificial spawning nests into three groups: early (first one-third), intermediate (middle one-third), and late (last one-third). These emergence groups were hatchery-reared separately (mono-culture) or together in a mixed population (co-culture) for approximately four months prior to the behavioural assays. Horizontal lines within each box represent median duration of escape attempts (s), ends of boxes represent the 25th and 75th percentiles, and whiskers represent the 10th and 90th percentiles. Open circles indicate outliers outside the 10th and 90th percentiles. The asterisks denote a significant difference at p < 0.05 (Tukey honestly significant difference). See Table 2 for sample sizes.

Regardless of previous rearing environment, the duration of the escape attempts in the net differed significantly among the three emergence groups (GLM; F = 3.133, df = 2, p = 0.047; Table 2; Fig. 2). Post hoc comparisons demonstrated that early emerging Atlantic salmon spend less time escaping compared to those with an intermediate emerging time (Tukey HSD test; p = 0.044), but no difference was detected between early and late emerging fry (Tukey HSD test; p = 0.204). The duration of escape attempts was also similar between intermediate and late emerging individuals (Tukey HSD test; p = 0.752).

PC activity and PC boldness was positively correlated with one another, indicating an activity–boldness syndrome (Spearman correlation; r _s_ = 0.375, p < 0.0001, n = 119). The duration of escape attempts during net restraining was not associated with either PC activity (Spearman correlation; r _s_ = 0.014, p = 0.882, n = 117) or PC boldness (Spearman correlation; r _s_ = −0.114, p = 0.123, n = 116).

### Performance in the stream

#### Survival

The survival rate of the Atlantic salmon in the experimental stream ranged from 50% to 70% among experimental treatment groups (Table 4). Mortality in the stream was likely caused by predation from mink and heron, which are common in the area. Starvation-related mortality is also thought to have occurred since ten fish were found dead in the stream from the 1^st^ of March to the 28^th^ of April 2013. These ten fish were in generally poor condition and did not have any obvious injuries, further supporting this assertion of starvation mortality. A binomial GLM revealed no significant effect of emergence time (GLM; LRT = 0.861, df = 2, p = 0.650), rearing environment (GLM; LRT = 2.258, df = 1, p = 0.133), length (GLM; LRT = 0.203, df = 1, p = 0.652), or the combined effect of emergence time and rearing environment (GLM; LRT = 0.309, df = 2, p = 0.857) on the probability of survival.

**Table 4:** Survival, migration, and specific growth rate (SGR) for Atlantic salmon (*Salmo salar*) among experimental treatment groups in the experimental stream.

Treatment	Survival (%)	Migration (%)	SGR (g day^−1^)
**Mono-culture**			
Early	70 (14)	65 (13)	0.0025 ± 0.0003 (14)
Intermediate	60 (12)	35 (7)	0.0034 ± 0.0006 (12)
Late	70 (14)	40 (8)	0.0036 ± 0.0006 (14)
**Co-culture**			
Early	60 (12)	40 (8)	0.0032 ± 0.0006 (12)
Intermediate	50 (10)	45 (9)	0.0027 ± 0.0005 (10)
Late	50 (10)	40 (8)	0.0032 ± 0.0003 (10)

Values are mean ± SE for SGR. Sample sizes are given in parentheses.

#### Growth

The mean specific growth rate (SGR; g day^−1^) of the Atlantic salmon was not related to emergence time, rearing environment, or their interaction (GLM; F ≤ 0.702, df = 1–2, p ≥ 0.500; Table 4), however, smaller fish generally had higher SGR than larger individuals (GLM; F = 10.352, df = 1, p = 0.002).

#### Migration status

In total, 53 of 120 fish were captured in the downstream trap, giving an overall migration rate of 44% (Table 4). These downstream migrations occurred from the 8^th^ of May to the 3^th^ of June 2013. There was a peak in the downstream descent that occurred from the 17^th^ of May to the 30^th^ of May, wherein 75% of the migrating fish were found in the trap. Although the rate of migration was particularly high for early emerging fry that were reared separately (65%), the analysis revealed no significant effect of emergence time (GLM; LRT = 1.638, df = 2, p = 0.441), rearing environment (GLM; LRT = 0.331, df = 1, p = 0.565), or their interaction (GLM; LRT = 2.759, df = 2, p = 0.252) on the probability of migration. Migration propensity was positively related to length (GLM; LRT = 5.864, df = 1, p = 0.016).

### Behaviour and performance in the stream

The probability of survival and migration in the stream was not related to PC activity, PC boldness, or duration of escape attempts during net restraining (GLM; LRT ≤ 0.575, df = 1, p ≥ 0.448). Furthermore, SGR was not associated with these behavioural measures (Pearson’s correlation; p ≥ 0.422).

## Discussion

### Behaviour

In the present study, the basal locomotor activity was positively correlated with boldness across experimental treatment groups, supporting observations that behavioural syndromes develop early in life of salmonid fishes [33,34]. The noted relationship between boldness and activity is similar to what has been reported in other fishes [48,56]. There was also a relationship between timing of emergence from the artificial spawning nests and behavioural response to the net restraining test. Fry with an early emergence time spent less time on escape attempts in the net compared to intermediate individuals (Fig. 2). The net restraining test has previously been used to discriminate individuals with contrasting stress coping styles [44,49,50]. For example, Gilthead seabream (*Sparus aurata*) that exhibit high activity during net restraining trials share traits characteristics of the proactive stress coping style, including greater risk-taking behaviour and fight-flight response to challenges [44]. If this relationship is present in Atlantic salmon, our results suggest that individuals with an intermediate emergence time have greater resemblance to the proactive coping style than early emerging conspecifics. This interpretation, however, is in contrast to earlier studies of wild populations of Atlantic salmon and selected rainbow trout strains, which have indicated a link between early emergence and the proactive stress coping style [25,26,28]. Moreover, basal activity and boldness were not related to emergence time, raising questions about the link between larval development rate and stress coping styles in this population of Atlantic salmon. Hence, an alternative explanation for the longer escape duration in fish with an intermediate emergence time might be that these individuals have higher physical stamina. The fact that the net escape response behaviour was unrelated to the activity–boldness syndrome also suggests that escape behaviour in Atlantic salmon represent a separate behavioural dimension. This adds to the view that suites of behavioural traits may be organized in independent dimensions [14,57]. Alternatively, this result might have to do with the chosen metric and other escape response metrics (e.g., predator escape) might correlate with the activity–boldness syndrome. Further studies are required to verify whether these behavioural axes remain stable over time and across different situations in nature, as previously demonstrated in other fish species [1].

Social interactions with conspecifics can affect behavioural traits of an individual [40–42]. For instance, bold rainbow trout may reduce their boldness after observing shy demonstrators [41], and European perch can adjust their level of boldness and exploration tendency to that of their social group [40]. In the present study, however, we could not detect any effect of co- or mono-rearing on the behaviour displayed by individuals with different emergence time. Earlier studies have suggested that social effects on behavioural development may be density-dependent, and it is possible that the relatively high rearing densities in this study (∼250 fish m^−2^) might not have facilitated social effects on behavioural expression [58,59]. Social behaviour is facilitated by the development of familiarity with other individuals over time, which in turn is limited by the number of individual identities that can be learned or memorized [60,61]. Hence, in a high-density environment there is little scope to develop social relations with specific individuals, which may have impaired the effects of social environment on behavioural development in our study [58].

### Performance in the stream

The results of the present study showed no significant effects of emergence time from artificial spawning nests and social rearing environment on growth, survival, and migration status of Atlantic salmon in the experimental stream. However, it is possible that due to the relatively low statistical power in this study we might have been unable to detect some correlations between performance in the stream and experimental treatment groups. For instance, albeit non-significant, it is worth noting that the downstream migration rate was 1.4 to 1.9 times higher for fish with an early emergence that were reared in mono-culture compared to the other treatments (Table 4). Similar results was reported by Metcalfe and Thorpe [25], demonstrating that a higher proportion of early emerging Atlantic salmon fry reached body size thresholds for smoltification during their first summer in the hatchery compared to those emerging later. This was ascribed to be a result of increased propensity for being socially dominant and higher metabolic rates among early emerging fry [25,26]. It should be noted that fish with different emergence times were reared in the same hatchery tank in the study by Metcalfe and Thorpe [25]. Taken together, these results indicate a link between migration status, emergence time, and social rearing environment, and we advocate more studies addressing this issue.

Although the size range of fish in this study was relatively small (i.e., 88 to 112 mm FL), body length at release into the stream was negatively correlated with SGR and positively correlated with migration propensity in the stream, consistent with previous studies on salmonids [62]. Our results also show that length was negatively related with boldness, suggesting that smaller fish were bolder than larger ones. Because smaller salmonids generally have higher mass specific metabolic rate and smaller energy reserves [63], and hungrier individuals are usually more willing to take risks [64,65], increased feeding motivation may have made smaller individuals appear bolder in the novel object test.

Previous studies on fitness (i.e., growth and survival) and life-history consequences of behavioural traits in salmonids have generated inconsistent results [1,30]. While some studies have demonstrated a link between behaviour, growth, and survival [33–35], others challenge the view of such relationships [36,37], emphasizing that the consequences of a given behavioural type on individual growth and survival are likely to differ across environmental context [1]. Although individual fish showed considerable variation in basal activity, boldness, and their escape response during net restraining in the current investigation, none of these behaviours were associated with growth, survival, or migration status under near-natural conditions. Since neither the persistence of behavioural expression over time and across context was examined here, context specificity or instability of behavioural traits cannot be ruled out as a possible explanation for the absence of a relationship between performance in the experimental stream and individual behavioural variation. For instance, unstable behavioural types could emerge if individual behavioural profiles measured in the hatchery were different from those expressed in the stream. It should be noted that we were also restricted in our sample population to fish larger than 85 mm FL among our emergence groups due to potential adverse effects of PIT-tagging on fish growth and survival [47]. Because of this limitation, it is possible that we omitted or reduced representation of certain behavioural phenotypes in our study, which might have contributed to the missing link between behaviour and performance in the stream. Hence, the present results add to the view that the link between behavioural expression from laboratory measures and subsequent performance in nature is often flexible and probably dependent on specific environmental conditions [1,30,66]. Additional empirical studies are needed to clarify the relationship between behavioural traits, fitness (i.e., growth and survival), and life-history. These studies should preferably be performed in a range of different natural habitats to elucidate whether and why some behavioural types are better suited to a given environment than others. From a more applied perspective, our results highlight the challenges associated with incorporating individual variation in behavioural traits into fisheries management and conservation.
